# Healthier Lifestyles Attenuated Association of Single or Mixture Exposure to Air Pollutants with Cardiometabolic Risk in Rural Chinese Adults

**DOI:** 10.3390/toxics10090541

**Published:** 2022-09-17

**Authors:** Xueyan Wu, Xiaotian Liu, Wei Liao, Xiaokang Dong, Ruiying Li, Jian Hou, Zhenxing Mao, Wenqian Huo, Yuming Guo, Shanshan Li, Gongbo Chen, Chongjian Wang

**Affiliations:** 1 Department of Epidemiology and Biostatistics, College of Public Health, Zhengzhou University, Zhengzhou 450001, China; 2 Department of Epidemiology and Preventive Medicine, School of Public Health and Preventive Medicine, Monash University, Melbourne, VIC 3010, Australia; 3 Guangdong Provincial Engineering Technology Research Center of Environmental and Health Risk Assessment, Department of Occupational and Environmental Health, School of Public Health, Sun Yat-sen University, Guangzhou 510080, China

**Keywords:** air pollutants, cardiometabolic risk, healthy lifestyle score, rural population

## Abstract

There is little research on how long-term exposure to independent and multiple air pollutants changes cardiometabolic risk in adults. In addition, previous studies focused on only the effect of one or two lifestyles on cardiometabolic risk. The evidence on the interactive effects of the lifestyle score and exposure to independent and mixtures of air pollutants on cardiometabolic risk is lacking. A total of 33,638 rural residents were included in the cross-sectional study. The three-year average concentrations of air pollutants for participants were predicted by using a satellite-based prediction. The air pollution score was created to assess the combined exposure of four air pollutants (PM_1_, PM_2.5_, PM_10_, and NO_2_). A gender−age-specific cardiometabolic risk score was calculated. Multivariable-adjusted linear regression and quantile g-computation were used to investigate the associations between air pollutants and cardiometabolic risk. Interaction plots were applied to describe the interactive effects of air pollution and the healthy lifestyle score on cardiometabolic risk. Per interquartile range (IQR) unit increases in PM_1_, PM_2.5_, PM_10_, or NO_2_ were associated with 0.162 (95% CI: 0.091, 0.233), 0.473 (95% CI: 0.388, 0.559), 0.718 (95% CI: 0.627, 0.810), and 0.795 (95% CI: 0.691, 0.898) unit increases in cardiometabolic risk score (all *p* < 0.05), respectively. A 0.854 (95% CI: 0.768, 0.940) unit increase in cardiometabolic risk was associated with each IQR increase in air pollution score. Furthermore, the strengths of associations of PM_1_, PM_2.5_, PM_10_, NO_2_, and the air pollution score on cardiometabolic risk score were attenuated with the healthy lifestyle score increase. In addition, there was no statistical significance after the lifestyle score equal to four scores for the effect of PM_1_ on the cardiometabolic risk score. In conclusions, individual or joint air pollutants were associated with an increased cardiometabolic risk. Improving the healthy lifestyle may be an effective method to improve cardiometabolic health in highly polluted rural regions.

## 1. Introduction

Cardiometabolic diseases, such as type 2 diabetes mellitus (T2DM) and cardiovascular disease (CVD), are having a growing impact worldwide, which are the leading cause of death globally [1]. In addition, over three-quarters of CVD deaths occur in low- and middle-income countries [1,2]. Due to rapid economic growth, air pollution has become a significant public health problem in China [3]. During 2013–2014, China’s population-weighted annual average PM_2.5_ concentrations were nearly seven times higher than the yearly average levels recommended by the World Health Organization (WHO) [4]. Despite the implementation of actions to improve air quality, PM_2.5_ concentrations are about six times the WHO guideline by 2017 [4]. Simultaneously, the increased risk of cardiometabolic diseases in response to exposure to air pollutants requires more attention. Studies have indicated that air pollution increases the morbidity and mortality of cardiovascular diseases [5,6], obesity [7], T2DM [8], dyslipidemia [9], hypertension [10,11], and metabolic syndrome [12]. However, most studies have focused on cardiometabolic-related diseases that have already occurred. Little attention has been paid to the risk state before disease onset. Previous studies have proposed metabolic syndrome, a group of cardiometabolic risk factors including abdominal obesity, dyslipidemia, elevated blood pressure, and high glucose concentrations [13]. In contrast to using dichotomous metabolic syndrome characteristics, a continuous cardiometabolic risk score increases statistical power [14,15]. Limited evidence exists examining how long-term exposure to independent and multiple air pollutants exposures ambient air pollutants affect changes in cardiometabolic risk in adults.

In addition, several behavioral factors are associated with an increased risk of cardiometabolic disease. There is growing evidence that lifestyles [12,16] might impact the relationship between air pollution and cardiometabolic disease. However, previous studies focused on only one or two lifestyles, and there is a lack of evidence on the interaction of lifestyles and exposure to independent and joint air pollutants on cardiometabolic risk in rural areas.

Hence, the primary aim of this study is to investigate the associations between independent and mixture exposure of ambient air pollutants and cardiometabolic risk using the cardiometabolic risk score and further explore the impact of the healthy lifestyle score on the association between air pollutants and cardiometabolic risk among rural Chinese adults.

## 2. Methods

### 2.1. Study Design and Participants

The participants of this study were from the baseline of the Henan Rural Cohort Study carried out in five rural regions (Suiping County, Yuzhou County, Xinxiang County, Tongxu County, and Yima County) of Henan Province in China between July 2015 and September 2017 (Figure S1). More details of the cohort about the study design, data collection, and measurements have been described elsewhere [17]. In short, this rural-based study incorporated a total of 39,259 people aged 18–79 by a multi-stage stratified cluster sampling method with a response rate of 93.7%. After excluding participants with cancer (*n* = 332), coronary heart disease (*n* = 1734), stroke (*n* = 2643), and participants with missing data on T2DM (*n* = 63), hypertension (*n* = 35), dyslipidemia (*n* = 52), and cardiometabolic risk predictors (*n* = 876) (including body mass index (BMI), waist circumference (WC), systolic blood pressure (SBP), fasting plasma glucose (FPG), diastolic blood pressure (SDP), triglyceride (TG), high-density lipoprotein cholesterol (HDLC), and the homeostasis model assessment 2 of insulin resistance (HOMA2-IR)), and who were omitted information needed (*n* = 335), a total of 33,638 participants were involved for further analysis.

### 2.2. Assessment of Air Pollution and the Air Pollution Score

Based on satellites data and the good predictive power of the estimated air pollutants concentrations that were validated, air pollutants (including particulate matter with an aerodynamic diameter of ≤1.0 µm (PM_1_), ≤2.5 µm (PM_2.5_), or ≤10 µm (PM_10_), as well as nitrogen dioxide (NO_2_)) were assessed using machine learning algorithms (random forests) for each participant [18,19]. Daily concentrations of PM_1_, PM_2.5_, PM_10_, and NO_2_ were calculated using a 0.1° × 0.1° spatial resolution. In addition to satellite-based data, meteorological (such as relative humidity, barometric pressure, and wind speed) and land use data (percentage of urban cover and greenness) were also incorporated into the model to estimate air pollutants. Three-year average concentrations of PM_1_, PM_2.5_, PM_10_, and NO_2_ before the baseline survey were calculated for all participants using the geocoding (longitude and latitude) of their corresponding home addresses. Additional details on air pollution exposure assessment are available in Supplementary Materials.

The air pollution score was used to assess the combined exposure to the four air pollutants [20]. Multivariate-adjusted risk estimates (*β* coefficients) for cardiometabolic risk were weighted in this study. The *β* coefficients were obtained from a final model with individual air pollutants as independent variables. The equation was written as:

(1) $$\begin{array}{c} \mathrm{Air}\;\mathrm{pollution}\;\mathrm{score}\;=\left(\beta_{{\mathrm{PM}}_{1}}\times {\mathrm{PM}}_{1}+\beta_{{\mathrm{PM}}_{1-2.5}}\times {\mathrm{PM}}_{1-2.5}+\beta_{{\mathrm{PM}}_{2.5-10}}\times {\mathrm{PM}}_{2.5-10}+\beta_{{\mathrm{NO}}_{2}}\times {\mathrm{NO}}_{2}\right)\\ \times (4/(\beta_{{\mathrm{PM}}_{1}}+\beta_{{\mathrm{PM}}_{1-2.5}}+\beta_{{\mathrm{PM}}_{2.5-10}}+\beta_{{\mathrm{NO}}_{2}})) \end{array}$$

where PM_1–2.5_ means particulate matter with 1.0 µm < an aerodynamics diameter ≤ 2.5 µm and PM_2.5–10_ means particulate matter with 2.5 µm < an aerodynamics diameter ≤ 10 µm.

The air pollution score ranged from 107.38 to 135.44, with higher scores indicating higher exposure to ambient air pollution.

### 2.3. Assessment of Cardiometabolic Risk

The cardiometabolic risk was assessed by a continuous cardiometabolic risk score [14], which was calculated by summing the standardized scores which were sex- and age-specific for BMI, WC, SBP, DBP, FPG, HOMA2-IR [21,22], TG, and HDLC (inverse ratio) based on the previous research examining cardiometabolic risk [23]. Considering that cardiometabolic-related indicators in patients with diabetes, hypertension, and dyslipidemia might be influenced by medication or other factors, the presence or absence of disease z-score was added to the calculation of the cardiometabolic risk score. The cardiometabolic risk score was constructed according to the previously published score made by Viitasalo et al. [23], who demonstrated its association with the incident of T2DM and major components of cardiovascular disease events in children and adults. The higher the cardiometabolic risk score, the greater the cardiometabolic risk [24].

The cardiometabolic risk score was calculated for each participant as follows:

(2) $$\begin{array}{c} \mathrm{Cardiometabolic}\;\mathrm{risk}\;\mathrm{score}\\ \;\;\;\;\;\;\;\;\;\;\;\;\;\;\;\;\;\;\;\;\;\;\;\;\;\;\;\;\;\;=\left[\left(\mathrm{zBMI}\;+\mathrm{zWC}\right)/2]+[\left(\mathrm{zSBP}\;+\;\mathrm{zDBP}\right)/2\right]+\mathrm{zFPG}+\mathrm{zHOMA}2\_\mathrm{IR}-\mathrm{zHDLC}+\mathrm{zTG}\;\\ \;\;\;\;\;\;\;\;\;\;\;\;\;\;\;\;\;\;\;\;\;\;\;\;\;\;\;\;\;\;+\mathrm{zT}2\mathrm{DM}+\mathrm{zHypertension}+\mathrm{zDyslipidemia} \end{array}$$

The cardiometabolic risk score ranged from −12.10 to 14.70, with higher scores indicating higher exposure to ambient air pollution.

This approach provided a continuous risk score with improved statistical power compared to a dichotomous metabolic syndrome. More information on the score and why it was chosen on details of the score was more thoroughly explained elsewhere [14,15].

### 2.4. Assessment of the Healthy Lifestyle Score

Lifestyle factors included smoking status, drinking status, physical activity, and diet. Smoking status was classified into low risk (never smoking or quitting for at least six months for reasons other than illness) and high risk (current smokers or quitters who had stopped smoking because of illness or exposure to passive smoking) [25], which were scored as 1 and 0, respectively. Alcohol drinking status was categorized into low-risk (no alcohol consumption or ≤25 g/day for men and ≤15 g/day for women) and high-risk (>25 g/day for men and >15 g/day for women were defined as high risk) [26,27], which were scored as 1 and 0, respectively. According to the International Physical Activity Questionnaire (IPAQ), physical activity was categorized based on the metabolic equivalent of task (MET) per minute per week in low, moderate, and high activity [28]. High physical activity and moderate physical activity were low risk and scored as 1. Light physical activity was high risk and scored as 0. The diet quality score was built based on food frequency question FFQ data availability [29]. Diet quality was assessed according to the Chinese Healthy Eating Index (CHEI) [30]. The highest score was 36, and for this study, a score of ≥22 was defined as low risk, and a score of <22 was defined as high risk (high risk = 0; low risk = 1) (Table S1) [31]. The detailed definition of the healthy lifestyle score was summarized in Table S2. The healthy lifestyle score ranged from 0 to 4, and a higher score indicated a healthier lifestyle. The detailed procedure was described in previous studies [31,32].

### 2.5. Statistical Analysis

Data were presented as mean ± standard deviation (SD) for continuous variables and count (%) for categorical variables. The student’s t-test or chi-square test was used to compare the participants’ baseline characteristics. Linear regression was used to analyze the associations between the independent and mixture exposure of ambient air pollutants and the cardiometabolic risk score. The three models were as follows: model 1—adjusted for age, gender, marital status, educational level, and personal averaged monthly income; model 2—adjusted as in model 1 plus smoking status, alcohol intake, and the diet score; model 3—adjusted as in model 2 plus family history of coronary heart disease, family history of stroke, family history of T2DM, and family history of hypertension. We performed subgroup analyzes on several factors: physical activity, the diet score, smoking status, and drinking status. The multiplicative interactions were performed to test the effect modification in subgroup analyzes by the generalized linear model, including the main effects of them and the product term. In addition, whether the association between air pollutants and cardiometabolic risk was modified by the healthy lifestyle score was tested. The sensitivity analysis of the associations of independent air pollutants or the air pollution score with cardiometabolic risk fixed at a 5th–95th percentile range was conducted. In addition, we assessed the association between these four mixed air pollutants and cardiometabolic risk using quantile g-computation [33] to further test the stability of the results. All data were analyzed by SPSS version 26 (IBM, Armonk, NY, USA) and R version 4.0.0. Statistical significance was set *p* < 0.05 at two tails.

## 3. Results

### 3.1. Basic Characteristics of the Study Population

Table 1 presents the basic demographic characteristics of the 33,638 (13,008 men and 20,630 women) participants aged 18–79 according to the quartiles of the air pollution score. Participants with a higher air pollution score were more likely to be older, to have lower levels of educational, personal averaged monthly income, and physical activity, to be with higher levels of BMI, WC, SBP, DBP, and FPG, and to have a higher prevalence of T2DM, hypertension, and dyslipidemia (all *p* < 0.001). Additionally, baseline characteristics of the study participants to the quartiles of the cardiometabolic risk score are presented in Table S3. Participants with a higher cardiometabolic risk score were likely to be older, have lower levels of physical activity, be with higher levels of BMI, WC, SBP, DBP, FPG, TG, and INS and have a higher prevalence of T2DM, hypertension, and dyslipidemia (all *p* < 0.05). Moreover, ambient air pollutants (PM_1_, PM_2.5_, PM_10_, and NO_2_) and the air pollution score were meaningfully different among the quartiles of the cardiometabolic risk score (all *p* < 0.001 and all *p* for trend ≤ 0.001) (Table S4). Table S5 exhibits the distributions of PM_1_, PM_2.5_, PM_10_, NO_2_, and the air pollution score. The means concentrations of PM_1_, PM_2.5_, PM_10_, and NO_2_ were 57.53μg/m^3^, 73.49 μg/m^3^, 132.60 μg/m^3^, and 39.95 μg/m^3^, respectively, with IQRs of 3.94 μg/m^3^, 4.57 μg/m^3^, 11.06 μg/m^3^, and 7.76 μg/m^3^, respectively. The air pollution score ranged from 107.38 to 135.44, with an IQR of 10.93. The cardiometabolic risk score ranges from −12.10 to 14.70, with an IQR of 6.17. The Pearson correlation coefficients among the air pollutants and air pollution score are shown in Table S6.

### 3.2. Associations of Single Air Pollution and the Air Pollution Score with Cardiometabolic Risk

Positive relationships were observed between air pollutants and the cardiometabolic risk score in all models (Table 2). After adjusting for potential confounding factors, per IQR unit increases in PM_1_, PM_2.5_, PM_10_, and NO_2_ were associated with 0.162 (95% CI: 0.091, 0.233), 0.473 (95% CI: 0.388, 0.559), 0.718 (95% CI: 0.627, 0.810), and 0.795 (95% CI: 0.691, 0.898) unit increases in cardiometabolic risk score, respectively. When jointly considering the four air pollutants (PM_1_, PM_2.5_, PM_10_, and NO_2_) by the air pollution score, there also was a significant association between higher levels of the air pollution score and the greater cardiometabolic risk score (0.854 (95% CI: 0.768, 0.940)). Sensitivity analyses (Tables S7 and S8) of these associations were further conducted by fixing the levels of PM_1_, PM_2.5_, PM_10_, NO_2_, and the air pollution score at the 5th and 95th ranges. After adjusting for potential confounding factors, per IQR unit increases in PM_1_, PM_2.5_, PM_10_, NO_2_, and the air pollution score were associated with 0.444 (95% CI: 0.352, 0.537), 0.721 (95% CI: 0.619, 0.822), 0.932 (95% CI: 0.826, 1.037), 0.998 (95% CI: 0.880, 1.115), and 0.943 (95% CI: 0.840, 1.046) unit increases in cardiometabolic risk score, respectively, and the strength of the association was very similar to that found in Table 2, which confirmed the consistency and robustness of our results. Moreover, the results were stable, when we assessed the association between these four mixed air pollutants and cardiometabolic risk using qg-computation (Table S8). Table S9 shows the associations between air pollutants, the air pollution score, and the cardiometabolic risk score by basic demographic characteristics. Interestingly, for the associations of air pollutants with the cardiometabolic risk score, PM_1_, PM_2.5_, PM_10_, NO_2_, and the air pollution score were significantly associated with the higher cardiometabolic risk score in the elderly (age: ≥65 years), men, or participants with a lower educational level and a higher averaged monthly income.

### 3.3. Associations of the Lifestyle Score with Cardiometabolic Risk

Table S10 shows that after adjusting for potential confounding factors, each one score increase in healthy lifestyle score was associated with a 0.174 (95% CI: −0.230 to −0.118) unit decrease in cardiometabolic risk score. Comparing with 0 score, the cardiometabolic risk scores were decreased by 0.591, 0.739, and 0.817 units for healthy lifestyle scores of 2, 3, and 4, respectively (all *p* < 0.05).

### 3.4. Associations of Air Pollutants and the Air Pollution Score with Cardiometabolic Risk by Lifestyle Factors

**Figure 1:**
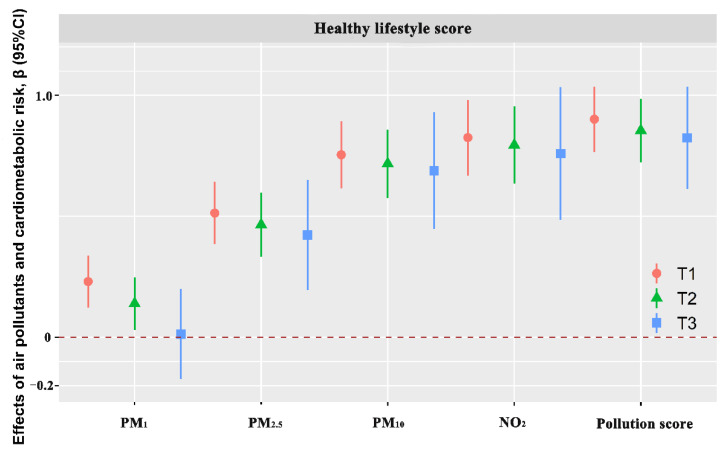
The associations of ambient air pollutants (per IQR increment) and the air pollution score (per IQR increment) with the cardiometabolic risk score by tertiles groups of healthy lifestyles. Abbreviations: PM_1_, particulate matter with an aerodynamics diameter of ≤1.0 µm; PM_2.5_, particulate matter with an aerodynamics diameter of ≤2.5 µm; PM_10_, particulate matter with an aerodynamics diameter of ≤10 µm; NO_2_, nitrogen dioxide. Concentrations of long-term exposure to four air pollutants were reflected by three-year averaged concentrations before the baseline of this study. The associations were adjusted for age, gender, marital status, educational level, personal averaged monthly income, family history of CHD, family history of stroke, family history of T2DM, and family history of hypertension.

Table S11 shows the associations between air pollutants, the air pollution score, and the cardiometabolic risk score in different subgroups of lifestyle factors. The four air pollutants and the air pollution score were significantly associated with a higher cardiometabolic risk score in high risk of smoking and drinking. In addition, the interactions of smoking were presented in associations between PM_1_ and the cardiometabolic risk score (*p*_interaction_ = 0.008). The interactions of drinking were presented in associations between PM_1_, PM_2.5_, PM_10_, NO_2_, and the air pollution score and the cardiometabolic risk score (all *p*_interaction_ < 0.01). The interactions of diet were presented in associations between PM_2.5_, PM_10_, and NO_2_ and the cardiometabolic risk score (all *p*_interaction_ < 0.05). The analyses of subgroups grouped by the tertiles of the healthy lifestyle score are presented in Figure 1 and Table S12. The estimated effects of the four air pollutants and the air pollution score on the cardiometabolic risk score were lowest in the highest tertile of the healthy lifestyle score, and the deleterious effect decreased with the tertile increase of the healthy lifestyle score.

### 3.5. Interactive Effect of the Lifestyle Score and Air Pollution on Cardiometabolic Risk

**Figure 2:**
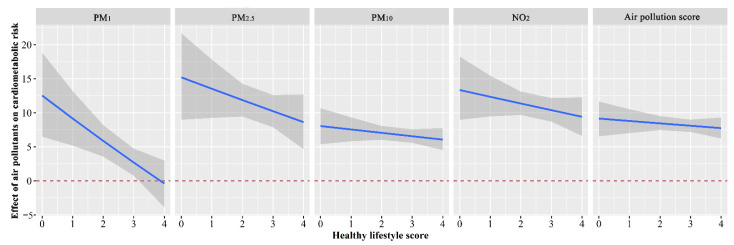
The estimated associations of ambient air pollutants (PM_1_, PM_2.5_, PM_10_, NO_2_, and the air pollution score) on the cardiometabolic risk score as a function of the healthy lifestyle score were analyzed using generalized linear models. Models were adjusted for age, gender, marital status, educational level, personal averaged monthly income, family history of CHD, family history of stroke, family history of T2DM, and family history of hypertension. The *X*-axis represents the healthy lifestyle score. The *Y*-axis represents the estimated effects and the 95% confidence intervals of ambient air pollutants on the cardiometabolic risk score along with the altered healthy lifestyle score. The blue lines and the grey areas represent the estimated effect and the 95% confidence interval. Abbreviation: PM_1_, particulate matter with an aerodynamics diameter of ≤1.0 µm; PM_2.5_, particulate matter with an aerodynamics diameter of ≤2.5 µm; PM_10_, particulate matter with an aerodynamics diameter of ≤10 µm; NO_2_, nitrogen dioxide. Concentrations of long-term exposure to four air pollutants were reflected by three-year averaged concentrations before the baseline of this study.

Compared to individuals with T3 levels of the healthy lifestyle score, T1 of the healthy lifestyle score was related to increased cardiometabolic risk accompanied by increased levels of PM_1_, PM_2.5_, PM_10_, and the air pollution score, whereas no significant interactive effect between T2 of the healthy lifestyle score and the single air pollutant or the air pollution score on cardiometabolic risk was observed (Figure S2). Figure 2 shows how the healthy lifestyle score decreases modified the effects of PM_1_, PM_2.5_, PM_10_, NO_2_, and the air pollution score on the cardiometabolic risk score, and the results indicated that the adverse effects of PM_1_, PM_2.5_, PM_10_, NO_2_, and the air pollution score on the cardiometabolic risk score were attenuated with the healthy lifestyle score increase. Moreover, there was no statistical significance after the lifestyle score equal to four scores for the effect of PM_1_ on the cardiometabolic risk score.

## 4. Discussion

In this study, we investigated the associations of independent and their combinations of ambient air pollutants on cardiometabolic risk and further explored the interaction of the healthy lifestyle score and air pollutants on cardiometabolic risk among rural Chinese adults. The results of the present study suggested that individual or joint air pollutants PM_1_, PM_2.5_, PM_10_, and NO_2_, were associated with an increased cardiometabolic risk. Moreover, a lower healthy lifestyle score aggravated a positive effect of long-term exposure to independent or mixture of air pollutants on cardiometabolic risk, implying that individuals with a low unhealthy lifestyle level might be more susceptible to adverse effects of exposure to high levels of individual or joint air pollutants on cardiometabolic health.

Although no studies directly analyzed the relationship between air pollutants and the cardiometabolic risk score, some animal investigations and epidemiological studies have elucidated the relationships between air pollutants and the individual components of metabolic syndrome. For example, some studies have shown that exposure to high concentrations of mixed air pollutants might increase the risk of obesity [7]. Long-term exposure to ambient PM_1_ PM_2.5_, PM_10_, and NO_2_ increased the risk of hypertension [10,11,34,35]. A population-based Swiss cohort found that both PM_10_ and NO_2_ were associated with the prevalence of diabetes [36]. A natural experiment in Beijing found that long-term exposure to air pollution particles increased the risk of metabolic syndrome [34]. A cross-sectional study also found that long-term exposure to ambient air pollutants was related to an increased risk of metabolic syndrome for rural Chinese adults [12]. Our study suggested that this increases in concentrations of PM_1_, PM_2.5_, PM_10_, and NO_2_ were associated with worsening cardiometabolic risk health, which was consistent with those studies. Meanwhile, the current study quantified how long-term exposure to independent air pollutant exposures affected changes in cardiometabolic risk among adults in rural populations. However, the relative studies were less, and the results were inconsistent. The PIAMA birth cohort did not find associations of air pollution with the cardiometabolic risk score in adolescents [37]. The GINIplus and LISAplus studies found that NO_2_, PM_2.5_, and PM_10_ were not associated with blood pressure in 2368 children [38]. Differences in population, sample size, study design, air pollutant measurements, and duration of exposure might be partly responsible for the inconsistent results of the limited studies. However, the mechanisms involved still need to be further explored.

In addition, a significant association was found between combined exposure to various air pollutants as assessed by the air pollution score and cardiometabolic risk. Humans are exposed to a complex mixture of air pollutants at the same time. The air pollution score could represent a more comprehensive exposure to air pollutants and recognize the importance of assessing the health burden caused by simultaneous exposure to multiple air pollutants [20]. This simple algorithm is also easy to interpret and promotes public health protection from air pollution [20]. Our research demonstrated that various air pollutants combined with exposure might collectively affect cardiometabolic risk. The mechanisms were not entirely understood. However, several mechanisms have been proposed to explain the phenomenon. Particulate matter exposure might induce oxidative stress and inflammation of organ tissues and the circulatory system [39]. Chronic exposure to PM_2.5_ leads to increased lipid deposition in adipose tissue [40] and insulin resistance [41]. Long-term exposure may be required to produce sustained inflammation and metabolic changes [34]. Exposure to ambient air containing high concentrations of PM_2.5_ induced TLR2/4-dependent inflammatory activation and lipid oxidation in the lungs, followed by systemic spillover, leading to metabolic disturbances [34]. However, these mechanisms were derived from animal model studies and need to be confirmed in humans.

Additionally, we further found an interaction between lifestyle and individual or joint air pollutants on cardiometabolic risk, implying that a higher concentration of air pollution and the unhealthier the lifestyle increased the risk of cardiometabolic diseases. Our previous research found that physical activity attenuated the effects of ambient air pollutants on the increased risk for metabolic syndrome [12] and cardiovascular disease [16]. Studies have shown that physical activity has beneficial effects on systemic inflammation [42] and that individuals who are physically active regularly might increase their resistance to the negative effects of air pollution [43]. Moreover, some studies have suggested a link between dietary effects on mitochondrial function [44] and possible modifications of dietary sources of antioxidants on air pollution and health effects [45]. In addition, some work has found synergetic effects of smoking and air pollution exposure on lung cancer risk [46] and chronic obstructive pulmonary disease [47]. Nevertheless, most studies mainly focused on one or two lifestyles separately. Few studies have shown the impact of integrating multiple lifestyles and exposure to high levels of ambient air pollutants on cardiometabolic risk. In this study, we created the healthy lifestyle score including diet, smoking, drinking, and physical activity. Our results showed that a healthy lifestyle might compensate somewhat for the deleterious effects on cardiometabolic health due to air pollution factors.

In this study, the annual average concentrations of PM_1_, PM_2.5_, PM_10_, and NO_2_ were found to be 57.54 μg/m^3^, 73.49 μg/m^3^, 132.60 μg/m^3^, and 39.96 μg/m^3^ in rural areas of China, respectively, which were much higher than the WHO recommendations air quality guideline levels, especially for PM_2.5_ (14.7 times) [48]. The study was conducted in rural areas of Henan Province, China, and used a satellite-based prediction approach to estimate historical air pollution exposures of rural adult populations. Familiar sources of rural air pollution involve household combustion equipment, motor vehicles, and industrial facilities. In addition, most households in rural Henan are one-story homes with kitchens, living areas, and courtyards [49], which may have better natural ventilation than urban homes. As the industry and the economy are developing rapidly, clean fuels and ventilation facilities are widely used in rural areas of China [50]. Rural residents have more daily outdoor activities and tend to more exposure to outdoor air pollution than urban residents. The satellite-based air pollutant estimates in this study were closely related to the participants’ exposure during the study period.

This study has some limitations. First, as these findings came from a cross-sectional study, a causative relationship between lifestyles, air pollutants, and cardiometabolic risk could not be established. However, the follow-up surveys of this cohort were ongoing, so that longitudinal data would be available to explore the associations further. Second, compared to urban environments, there might be a high level of spatial error in misclassification when geocoding rural addresses. Although the data on the lifestyles were obtained from face-to-face questionnaires administered by professionally trained interviewers, most were obtained from participant self-reports, with the potential for over- or underestimation, which might produce recall bias. Third, although important variables concerning cardiometabolic risk were already adjusted, potential confounding factors are still present in this study. Although these limitations exist, this large-scale population study might still reflect the associations between long-term exposure to ambient air pollution and cardiometabolic risk in some measures.

## 5. Conclusions

The study found that individual or joint air pollutants of PM_1_, PM_2.5_, PM_10_, and NO_2_, were associated with an increased cardiometabolic risk. Moreover, a lower healthy lifestyle score increased the strength of the associations of long-term exposure to independent or a mixture of air pollutants on cardiometabolic risk, implying that individuals with unhealthy lifestyles might be more susceptible to adverse effects of exposure to individual or joint air pollutants on cardiometabolic health.

## Figures and Tables

**Table 1:** Summary statistics of the characteristics for the participants according to the quartiles of the air pollution score.

Variable	Air pollution score				*p*
	Q1	Q2	Q3	Q4	
**Age (years)**	55.3 (11.82)	54.94 (12.30)	52.59 (13.20)	55.90 (11.52)	<0.001 ^a^
**Sex**					<0.001 ^b^
Men	3440 (41.84)	3493 (41.09)	3144 (38.12)	2931 (33.81)	
Women	4782 (58.16)	5008 (58.91)	5103 (61.88)	5737 (66.19)	
**Marital status**					<0.001 ^b^
Married/cohabitation	7422 (90.27)	7693 (90.50)	7506 (91.01)	7715 (89.01)	
Unmarried/divorced/widowed	800 (9.73)	808 (9.50)	741 (8.99)	953 (10.99)	
**Educational level**					<0.001 ^b^
Elementary school or below	3953 (48.08)	3882 (45.67)	2845 (34.50)	3879 (44.75)	
Middle school	3288 (39.99)	3342 (39.31)	3364 (40.79)	3711 (42.81)	
High school or above	981 (11.93)	1277 (15.02)	2038 (24.71)	1078 (12.44)	
**Personal averaged monthly income**					<0.001 ^b^
<500 RMB	3317 (40.34)	2451 (28.83)	2807 (34.04)	3048 (35.16)	
500–999 RMB	2424 (29.48)	2791 (32.83)	2818 (34.17)	3137 (36.19)	
≥1000 RMB	2481 (30.18)	3259 (38.34)	2622 (31.79)	2483 (28.65)	
**Current regular smokers**	1836 (22.33)	1805 (21.23)	1522 (18.46)	1339 (15.45)	<0.001 ^b^
**Current regular drinking**	1422 (17.30)	1486 (17.48)	1719 (20.84)	1628 (18.78)	<0.001 ^b^
**Physical activity**					<0.001 ^b^
Low	2242 (27.27)	2228 (26.21)	3225 (39.11)	2769 (31.95)	
Moderate	3583 (43.58)	3479 (40.92)	2443 (29.62)	3370 (38.88)	
High	2397 (29.15)	2794 (32.87)	2579 (31.27)	2529 (29.18)	
**Diet score**	19.71 (3.88)	20.58 (4.20)	21.99 (4.14)	19.57 (4.13)	<0.001 ^a^
**BMI (kg/m^2^)**	24.23 (3.37)	24.43 (3.54)	25.35 (3.54)	25.16 (3.48)	<0.001 ^a^
**WC (cm)**	80.85 (9.90)	83.38 (10.29)	85.99 (10.39)	85.15 (9.84)	<0.001 ^a^
**SBP (mmHg)**	119.07 (17.52)	124.77 (20.17)	128.75 (19.78)	126.97 (19.75)	<0.001 ^a^
**DBP (mmHg)**	73.03 (10.57)	76.93 (11.37)	80.69 (11.40)	78.68 (11.42)	<0.001 ^a^
**FPG (mmol/L)**	5.27 (1.12)	5.32 (1.28)	5.64 (1.32)	5.58 (1.42)	<0.001 ^a^
**TG (mmol/L)**	1.83 (1.14)	1.56 (1.03)	1.62 (1.08)	1.59 (1.03)	<0.001 ^a^
**HDLC (mmol/L)**	1.37 (0.34)	1.36 (0.33)	1.30 (0.33)	1.31 (0.33)	<0.001 ^a^
**INS (μIU/mL)**	13.10 (4.42)	11.23 (5.12)	8.58 (4.71)	9.99 (5.18)	<0.001 ^a^
**Family history of CHD (Yes)**	802 (9.75)	699 (8.45)	472 (5.74)	657 (7.58)	<0.001 ^b^
**Family history of Stroke (Yes)**	789 (9.60)	801 (9.68)	384 (4.67)	773 (8.92)	<0.001 ^b^
**Family history of hypertension (Yes)**	1319 (16.04)	1527 (17.96)	1746 (21.17)	1875 (21.63)	<0.001 ^b^
**Family history of T2DM (Yes)**	223 (2.71)	271 (3.19)	432 (5.24)	481 (5.55)	<0.001 ^b^
**T2DM (Yes)**	800 (9.73)	808 (9.50)	741 (8.99)	953 (10.99)	<0.001 ^b^
**Hypertension (Yes)**	435 (5.29)	536 (6.31)	781 (9.47)	852 (9.83)	<0.001 ^b^
**Dyslipidemia (Yes)**	1569 (19.08)	2351 (27.66)	3181 (38.57)	2915 (33.63)	<0.001 ^b^

Data are mean (SD) or N (%). BMI, body mass index calculated by using the weight divided by the square of the height of a person (kg/m^2^); WC, waist circumference; SBP, systolic blood pressure; DBP, diastolic blood pressure; FPG, fasting plasma glucose; TG, triglyceride; HDLC, high-density lipoprotein cholesterol; INS, insulin; CHD, coronary heart disease; T2DM, type 2 diabetes mellitus. ^a^, ANOVA tests was used to compare the mean difference of continuous variables by quartiles of cardiometabolic risk; ^b^, a chi-square test was used to test the distributions of categorical variables by quartiles of cardiometabolic risk.

**Table 2:** Estimated independent and air pollution scores of ambient air pollutants (per IQR increment) on cardiometabolic risk.

Air Pollutants	Line Regression *β* (95% CI)		
	Model 1	Model 2	Model 3
**PM_1_**	0.230 (0.159, 0.301) *	0.217 (0.146, 0.288) *	0.162 (0.091, 0.233) *
**PM_2.5_**	0.570 (0.484, 0.655) *	0.550 (0.464, 0.635) *	0.473 (0.388, 0.559) *
**PM_10_**	0.828 (0.736, 0.919) *	0.807 (0.715, 0.899) *	0.718 (0.627, 0.810) *
**NO_2_**	0.909 (0.806, 1.013) *	0.888 (0.784, 0.991) *	0.795 (0.691, 0.898) *
**Air pollution score**	0.952 (0.866, 1.038) *	0.933 (0.847, 1.020) *	0.854 (0.768, 0.940) *

*, *p* < 0.001. PM_1_, particulate matter with an aerodynamics diameter of ≤1.0 µm; PM_2.5_, particulate matter with an aerodynamics diameter of ≤2.5 µm; PM_10_, particulate matter with an aerodynamics diameter of ≤10 µm; NO_2_, nitrogen dioxide. Concentrations of long-term exposure to four air pollutants were reflected by three-year averaged concentrations before the baseline of this study. Model 1: adjusted for age, gender, marital status, educational level, and personal averaged monthly income; model 2: adjusted as in model 1 plus smoking status, alcohol intake, and the diet score; model 3: adjusted as in model 2 plus family history of coronary heart disease, family history of stroke, family history of T2DM, and family history of hypertension.

## Data Availability

The raw data supporting the conclusions of this manuscript will be made available by the authors, without undue reservation, to any qualified researcher.

## Supplementary Materials

The following supporting information can be downloaded at: https://www.mdpi.com/article/10.3390/toxics10090541/s1, Figure S1: Locations of the five survey sites in the Henan Rural Cohort; Figure S2: Changes in estimated associations of healthy lifestyle score on cardiometabolic risk score along with increased levels of single- air pollutant and air pollution score; Table S1: The classification and score of different diet score; Table S2: The classification and score of different lifestyle factors; Table S3: Baseline characteristics according to the quartiles of cardiometabolic risk score; Table S4: Median (inter-quartile range) of 3-year average concentrations of ambient air pollutants and the air pollution score according to the quartiles of cardiometabolic risk score; Table S5: 3-years average concentrations of ambient air pollutants, the air pollution score, and cardiometabolic risk score; Table S6: Pearson correlations between the individual air pollutant and air pollution score; Table S7: Estimated in-dependent and air pollution score (truncated at 5th and 95th percentiles) of ambient air pollutants (per IQR increment) on cardiometabolic risk in sensitivity analysis; Table S8: Estimated mixture of air pollutants (using quantile g-computation) on cardiometabolic risk in sensitivity analysis; Table S9: The associations of ambient air pollutants (per IQR increment) and air pollution score (per IQR increment) with cardiometabolic risk score (β, 95%CI) by basic demographic characteristics; Table S10: Estimated the effects of lifestyle score on cardiometabolic risk; Table S11: The associations of ambient air pollutants (per IQR increment) and air pollution score (per IQR increment) with cardiometabolic risk score (β, 95%CI) by lifestyle factors; Table S12: The associations of ambient air pollutants (per IQR increment) and air pollution score (per IQR increment) with cardiometabolic risk score (β, 95%CI) by healthy lifestyle score [18,19,51].
