# Supplementary material for: Healthier Lifestyles Attenuated Association of Single or Mixture Exposure to Air Pollutants with Cardiometabolic Risk in Rural Chinese Adults
Source: Toxics. 2022 Sep 17;10(9):541. doi: 10.3390/toxics10090541 (PMC9503940; doi:10.3390/toxics10090541)
Supplement: Supplementary file 1 [file toxics-10-00541-s001.zip › toxics-1813888-supplementary.pdf]

Supplementary captions

Supplementary Table S1. The classification and score of different diet score.

Supplementary Table S2. The classification and score of different lifestyle factors.

Supplementary Table S3. Baseline characteristics according to the quartiles of cardiometabolic risk score.

Supplementary Table S4. Median (interquartile range) of 3-year average concentrations of ambient air pollutants and the air pollution score according to the quartiles of cardiometabolic risk score.

Supplementary Table S5. 3-years average concentrations of ambient air pollutants, the air pollution score, and cardiometabolic risk score.

Supplementary Table S6. Pearson correlations between the individual air pollutant and air pollution score.

Supplementary Table S7. Estimated independent and air pollution score (truncated at 5th and 95th percentiles) of ambient air pollutants (per IQR increment) on cardiometabolic risk in sensitivity analysis.

Supplementary Table S8. Estimated mixture of air pollutants (using quantile g-computation) on cardiometabolic risk in sensitivity analysis.

Supplementary Table S9. The associations of ambient air pollutants (per IQR increment) and air pollution score (per IQR increment) with cardiometabolic risk score (*β*, 95%CI) by basic demographic characteristics.

Supplementary Table S10. Estimated the effects of lifestyle score on cardiometabolic risk.

Supplementary Table S11. The associations of ambient air pollutants (per IQR increment) and air pollution score (per IQR increment) with cardiometabolic risk score (*β*, 95%CI) by lifestyle factors.

Supplementary Table S12. The associations of ambient air pollutants (per IQR increment) and air pollution score (per IQR increment) with cardiometabolic risk score (β, 95%CI) by healthy lifestyle score.

Supplementary Figure S1. Locations of the five survey sites in the Henan Rural Cohort.

Supplementary Figure S2. Changes in estimated associations of healthy lifestyle score on cardiometabolic risk score along with increased levels of single- air pollutant and air pollution

score. Highest tertile of healthy lifestyle score was used as the reference group. The models were adjusted for age, gender, marital status, educational level, personal averaged monthly income, family history of CHD, family history of Stroke, family history of T2DM, family history of hypertension. The lines and corresponding shaded areas represented the estimated effect and 95% confidence interval of healthy lifestyle score on cardiometabolic risk along with increasing levels of single- air pollutant and air pollution score

Estimating concentrations of air pollutants

Supplementary Table S1. The classification and score of different diet score.

Food types	Frequency of food consumption					Largest score
	once/day	≥once/ week	≥once/month	≥once/ year	No consumption	
Red meat	0	1	2	3	4	4
Fish	4	3	2	1	0	4
Eggs	4	3	2	1	0	4
Milk products	4	3	2	1	0	4
Soy products	4	3	2	1	0	4
Nuts	4	3	2	1	0	4
Whole grains	4	3	2	1	0	4
Fruits	4	3	2	1	0	4
Vegetables	4	3	2	1	0	4
**Diet score**			0-36			

Supplementary Table S2. The classification and score of different lifestyle factors.

lifestyle	Definition	Score
**Smoking status**		
Low risk	Low risk was defined as not smoking or quitting for at least six months for reasons other than illness.	1
Hight risk	High risk was defined as current smokers or quitters who had stopped smoking because of illness, or exposure to passive smoking.	0
**Drinking status**		
Low risk	no alcohol consumption or ≤ 25 g/day for men and ≤ 15 g/day for women is defined as low risk	1
Hight risk	> 25 g/day for men and > 15 g/day for women is defined as high risk	0
**physical activity**	Physical activity (low, moderate and high) was assessed according to the International Physical Activity Questionnaire	
Low risk	Define heavy physical activity and moderate physical activity as low risk.	1
Hight risk	Define light physical activity as high risk.	0
**Diet**		
Low risk	diet score of ≥ 22 is defined as low risk.	1
Hight risk	diet score of < 22 is defined as high risk.	0
**Healthy lifestyle score**		0-4

Supplementary Table S3. Summary statistics of the characteristics for the participants according to the quartiles of cardiometabolic risk score.

Variables	Cardiometabolic risk score				*P*
	Q1	Q2	Q3	Q4	
**Age (years)**	54.60 (12.46)	53.69 (12.91)	55.04 (12.06)	55.58 (11.49)	<0.001[a]
**Sex**					<0.001[b]
Men	3647 (40.99)	3133 (36.18)	3189 (38.05)	3039 (39.46)	
Women	5250 (59.01)	5526 (63.82)	5192 (61.95)	4662 (60.54)	
**Marital status**					<0.001[b]
Married/cohabitation	7928 (89.11)	7822 (90.33)	7570 (90.32)	7016 (91.11)	
Unmarried/divorced/ widowed	969 (10.89)	837 (9.67)	811 (9.68)	685 (8.89)	
**Educational level**					0.002[b]
Elementary school or below	3953 (44.43)	3665 (42.33)	3565 (42.54)	3376 (43.84)	
Middle school	3621 (40.70)	3541 (40.89)	3482 (41.55)	3061 (39.75)	
High school or above	1323 (14.87)	1453 (16.78)	1334 (15.92)	1264 (16.41)	
**Personal averaged monthly income**					0.004[b]
< 500 RMB	3182 (35.76)	3038 (35.08)	2798 (33.39)	2605 (33.83)	
500–999 RMB	2860 (32.15)	2807 (32.42)	2875 (34.30)	2628 (34.13)	
≥1000 RMB	2855 (32.09)	2814 (32.50)	2708 (32.31)	2468 (32.05)	
**Current regular smokers**	2019 (22.69)	1580 (18.25)	1523 (18.17)	1380 (17.92)	<0.001[b]
**Current regular drinking**	1655 (18.60)	1522 (17.58)	1555 (18.55)	1523 (19.78)	0.001[b]
**Physical activity**					<0.001[b]
Low	2345 (26.36)	2628 (30.35)	2747 (32.78)	2744 (35.63)	
Moderate	3420 (38.44)	3344 (38.62)	3214 (38.35)	2897 (37.62)	
High	3132 (35.20)	2687 (31.03)	2420 (28.87)	2060 (26.75)	
**Diet score**	20.33 (4.13)	20.53 (4.25)	20.55 (4.19)	20.39 (4.24)	0.001[a]
**BMI (kg/m2)**	22.02 (2.51)	24.30 (2.80)	25.74 (2.88)	27.51 (3.38)	<0.001[a]
**WC (cm)**	75.74 (7.72)	82.24 (8.43)	86.76 (8.41)	91.89 (9.16)	<0.001[a]
**SBP (mmHg)**	112.96 (12.86)	121.39 (16.72)	129.48 (19.63)	137.75 (20.03)	<0.001[a]
**DBP (mmHg)**	69.39 (8.06)	75.23 (8.98)	80.28 (10.85)	85.73 (11.42)	<0.001[a]
**FPG (mmol/L)**	4.96 (0.50)	5.16 (0.56)	5.38 (0.82)	6.43 (2.16)	<0.001[a]
**TG (mmol/L)**	1.05 (0.37)	1.30 (0.50)	1.74 (0.80)	2.63 (1.55)	<0.001[a]
**HDLC (mmol/L)**	1.58 (0.30)	1.38 (0.27)	1.24 (0.27)	1.10 (0.27)	<0.001[a]
**INS (μIU/mL)**	8.73 (3.60)	10.04 (4.30)	10.98 (4.71)	13.49 (6.55)	<0.001[a]
**Family history of CHD (Yes)**	668 (7.56)	712 (8.29)	674 (8.10)	576 (7.54)	0.170[b]
**Family history of Stroke (Yes)**	763 (8.64)	695 (8.09)	676 (8.13)	613 (8.02)	0.442[b]
**Family history of hypertension (Yes)**	1193 (13.41)	1527 (17.63)	1795 (21.42)	1952 (25.35)	<0.001[b]
**Family history of T2DM (Yes)**	241 (2.71)	321 (3.71)	377 (4.50)	468 (6.08)	<0.001[b]
**T2DM (Yes)**	14 (0.16)	82 (0.95)	383 (4.57)	2125 (27.59)	<0.001[b]
**Hypertension (Yes)**	223 (2.51)	1557 (17.98)	3341 (39.86)	4895 (63.56)	<0.001[b]
**Dyslipidemia (Yes)**	244 (2.74)	1480 (17.09)	4138 (49.37)	6101 (79.22)	<0.001[b]

Data are mean (SD) or N (%).

BMI, body mass index, weight (kg)/height $(m)^2$; WC, waist circumference; SBP, systolic blood pressure; DBP, diastolic blood pressure; FPG, fasting plasma glucose; TG, triglyceride; HDLC, high-density lipoprotein cholesterol; INS, insulin; CHD, coronary heart disease; T2DM, type 2 diabetes mellitus.

[a], ANOVA tests was used to compare the mean difference of continuous variables by quartiles of cardiometabolic risk; [b], Chi-square test was used to test the distributions of categorical variables by quartiles of cardiometabolic risk.

Supplementary Table S4. Median (interquartile range) of 3-year average concentrations of ambient air pollutants and the air pollution score according to the quartiles of cardiometabolic risk score.

Air pollutants	Cardiometabolic risk score				P [a]	P_{trend}
	Q1	Q2	Q3	Q4		
PM_1 (μg/m^3)	56.81(55.48, 59.52)	56.96(55.51, 58.96)	57.36(56.01, 59.55)	57.53(56.18, 59.63)	<0.001	0.001
$PM_{2.5}$ (μg/m^3)	72.69(70.90, 76.05)	73.25(71.48, 76.03)	73.47(71.51, 76.08)	73.82(71.60, 76.10)	<0.001	<0.001
PM_{10} (μg/m^3)	129.92(125.99, 138.83)	132.63(127.83, 138.59)	133.60(128.03, 139.03)	134.60(128.28, 139.13)	<0.001	<0.001
NO_2 (μg/m^3)	37.63(35.96, 44.27)	40.11(36.27, 42.67)	40.67(36.78, 44.34)	40.73(37.27, 44.34)	<0.001	<0.001
Air pollution score	115.10(111.64, 122.61)	118.78(111.66, 122.63)	119.91(111.97, 122.74)	119.99(112.52, 123.11)	<0.001	<0.001

Data are Median (IQR). [a], ANOVA tests.

PM_1, particulate matter with an aerodynamics diameter ≤ 1.0 μm; $PM_{2.5}$, particulate matter with an aerodynamics diameter ≤ 2.5 μm; PM_{10}, particulate matter with an aerodynamics diameter ≤ 10 μm; NO_2, nitrogen dioxide. Concentrations of long-term exposure to four air pollutants were reflected by 3-year averaged concentrations before baseline of this study.

Supplementary Table S5. 3-years average concentrations of ambient air pollutants, the air pollution score, and cardiometabolic risk score.

Air pollutants	Mean ± SD	Median	Quantiles			IQR	Min	Max
			Q_{25}	Q_{50}	Q_{75}			
PM_1 (μg/m^3)	57.53±2.69	57.14	55.57	57.14	59.52	3.94	48.09	70.93
$PM_{2.5}$ (μg/m^3)	73.49±2.59	73.32	71.48	73.32	76.05	4.57	68.04	84.95
PM_{10} (μg/m^3)	132.59±5.84	133.10	127.84	133.10	138.90	11.06	122.36	148.78
NO_2 (μg/m^3)	39.95±3.63	40.29	36.50	40.29	44.27	7.76	31.00	49.81
Air pollution score	118.21±6.11	119.73	111.72	119.73	122.65	10.93	107.38	135.44
Cardiometabolic risk score	-0.34±4.53	-0.98	-3.77	-0.98	2.40	6.17	-12.10	14.70

PM_1, particulate matter with an aerodynamics diameter ≤ 1.0 μm; $PM_{2.5}$, particulate matter with an aerodynamics diameter ≤ 2.5 μm; PM_{10}, particulate matter with an aerodynamics diameter ≤ 10 μm; NO_2, nitrogen dioxide. Concentrations of long-term exposure to four air pollutants were reflected by 3-year averaged concentrations before baseline of this study.

Supplementary Table S6. Pearson correlations between the individual air pollutant and air pollution score.

Exposures	PM_1	$PM_{2.5}$	PM_{10}	NO_2	Air pollution score
**PM_1 (μg/m^3)**	1.000				
**$PM_{2.5}$ (μg/m^3)**	0.935*	1.000			
**PM_{10} (μg/m^3)**	0.802*	0.942*	1.000		
**NO_2 (μg/m^3)**	0.790*	0.902*	0.968*	1.000	
**Air pollution score**	0.533*	0.779*	0.929*	0.900*	1.000

[*], $P < 0.001$.

PM_1, particulate matter with an aerodynamics diameter ≤ 1.0 μm; $PM_{2.5}$, particulate matter with an aerodynamics diameter ≤ 2.5 μm; PM_{10}, particulate matter with an aerodynamics diameter ≤ 10 μm; NO_2, nitrogen dioxide. Concentrations of long-term exposure to four air pollutants were reflected by 3-year averaged concentrations before baseline of this study.

Supplementary Table S7. Estimated independent and air pollution score (truncated at 5th and 95th percentiles) of ambient air pollutants (per IQR increment) on cardiometabolic risk in sensitivity analysis.

Air pollutants	Line Regression *β* (95% CI)		
	**Model 1**	**Model 2**	**Model 3**
**PM_1**	0.538(0.445, 0.630)*	0.524(0.431, 0.616)*	0.444(0.352, 0.537)*
**$PM_{2.5}$**	0.824(0.722, 0.925)*	0.800(0.699, 0.902)*	0.721(0.619, 0.822)*
**PM_{10}**	1.047(0.942, 1.153)*	1.026(0.920, 1.132)*	0.932(0.826, 1.037)*
**NO_2**	1.114(0.996, 1.232)*	1.092(0.974, 1.210)*	0.998(0.880, 1.115)*
**Air pollution score**	1.057(0.854, 1.160)*	1.031(0.927, 1.134)*	0.943(0.840, 1.046)*

*, $P < 0.001$.

PM_1, particulate matter with an aerodynamics diameter ≤ 1.0 μm; $PM_{2.5}$, particulate matter with an aerodynamics diameter ≤ 2.5 μm; PM_{10}, particulate matter with an aerodynamics diameter ≤ 10 μm; NO_2, nitrogen dioxide. Concentrations of long-term exposure to four air pollutants were reflected by 3-year averaged concentrations before baseline of this study.

Model 1: adjusted for age, gender, marital status, educational level, and personal averaged monthly income; Model 2: adjusted as in model 1 plus smoking status, alcohol intake, and diet score; Model 3: adjusted as in model 2 plus family history of coronary heart disease, family history of Stroke, family history of T2DM, and family history of hypertension.

Supplementary Table S8. Estimated mixture of air pollutants (using quantile g-computation) on cardiometabolic risk in sensitivity analysis.

Models	Mixture of air pollutants by qg-computation	
	***β* (95% CI)**	***P***
Model 1	0.620(0.564, 0.676)	<0.001
Model 2	0.608(0.552, 0.664)	<0.001
Model 3	0.570(0.514, 0.626)	<0.001

qg-computation, quantile g-computation.

Model 1: adjusted for age, gender, marital status, educational level, and personal averaged monthly income; Model 2: adjusted as in model 1 plus smoking status, alcohol intake, and diet score; Model 3: adjusted as in model 2 plus family history of coronary heart disease, family history of Stroke, family history of T2DM, and family history of hypertension.

Supplementary Table S9. The associations of ambient air pollutants (per IQR increment) and air pollution score (per IQR increment) with cardiometabolic risk score (β, 95%CI) by basic demographic characteristics

Variables	PM_1	*P*	$PM_{2.5}$	*P*	PM_{10}	*P*	NO_2	*P*	Air pollution score	*P*
**Age**		0.125		0.005		0.004		0.097		0.002
< 65	0.136(0.055, 0.217)*		0.427(0.329, 0.525)*		0.677(0.572, 0.782)*		0.780(0.660, 0.900)*		0.818(0.719, 0.918)*	
≥ 65	0.272(0.122, 0.421)*		0.694(0.517, 0.870)*		0.934(0.748, 1.121)*		0.921(0.713, 1.128)*		1.065(0.890, 1.239)*	
**Sex**		<0.001		<0.001		<0.001		<0.001		<0.001
Men	0.324(0.207, 0.441)*		0.697(0.558, 0.837)*		0.981(0.831, 1.130)*		1.083(0.913, 1.253)*		1.112(0.970, 1.255)*	
Women	0.054(-0.035, 0.143)		0.329(0.221, 0.437)*		0.556(0.440, 0.672)*		0.624(0.493, 0.754)*		0.709(0.601, 0.817)*	
**Marital status**		0.400		0.977		0.639		0.921		0.234
Married/cohabitation	0.171(0.097, 0.246)*		0.482(0.392, 0.572)*		0.725(0.628, 0.821)*		0.810(0.700, 0.919)*		0.856(0.765, 0.947)*	
Unmarried/divorced/ widowed	0.021(-0.207, 0.249)		0.408(0.136, 0.681)*		0.702(0.412, 0.992)*		0.694(0.371, 1.016)*		0.931(0.659, 1.203)*	
**Educational level**		0.199		0.071		0.146		0.186		0.047
≤Primary school	0.211(0.099, 0.323)*		0.549(0.418, 0.680)*		0.773(0.634, 0.912)*		0.847(0.691, 1.003)*		0.930(0.798, 1.061)*	
> Primary school	0.130(0.039, 0.222)*		0.431(0.317, 0.544)*		0.701(0.579, 0.823)*		0.780(0.641, 0.919)*		0.826(0.712, 0.941)*	
**Personal averaged monthly income**		0.469		0.073		0.028		0.010		0.010
< 500 RMB	0.190(0.066, 0.313)*		0.552(0.406, 0.698)*		0.814(0.660, 0.969)*		0.914(0.740, 1.089)*		0.953 (0.810, 1.096)*	
500–999 RMB	0.126(0.006, 0.246)*		0.363(0.216, 0.510)*		0.566(0.407, 0.725)*		0.595(0.416, 0.775)*		0.676(0.524, 0.828)*	
≥1000 RMB	0.157(0.030, 0.283)*		0.515(0.361, 0.669)*		0.794(0.629, 0.959)*		0.890(0.702, 1.078)*		0.960 (0.805, 1.115)*	

P, *P* for interaction; *, $P < 0.05$.

PM_1, particulate matter with an aerodynamics diameter ≤ 1.0 µm; $PM_{2.5}$, particulate matter with an aerodynamics diameter ≤ 2.5 µm; PM_{10}, particulate matter with an aerodynamics diameter ≤ 10 µm; NO_2, nitrogen dioxide. Concentrations of long-term exposure to four air pollutants were reflected by 3-year averaged concentrations before baseline of this study.

Adjusted for age, gender, marital status, educational level, personal averaged monthly income, smoking status, alcohol intake, diet score. family history of CHD, family history of Stroke, family history of T2DM, and family history of hypertension (unless stratified by the respective factor).

Multiplicative interaction of air pollution and healthy lifestyles score on cardiometabolic risk score was assessed by including the main effects of them and the product term in the model and the $P_{interaction}$ was represented by the p-value of product term.

Supplementary Table S10. Estimated the effects of lifestyle score on cardiometabolic risk.

	β **(95% CI)**	***P***
**Healthy lifestyle score**	-0.174(-0.230, -0.118)	<0.001
**Categorical variables**		
0	0	
1	-0.242(-0.728, 0.245)	0.330
2	-0.591(-1.060, -0.122)	0.014
3	-0.739(-1.208, -0.270)	0.002
4	-0.817(-1.295, -0.339)	0.001

Adjusted for age, gender, marital status, educational level, personal averaged monthly income, family history of CHD, family history of stroke, family history of T2DM, and family history of hypertension.

Supplementary Table S11. The associations of ambient air pollutants (per IQR increment) and air pollution score (per IQR increment) with cardiometabolic risk score (*β*, 95%CI) by lifestyle factors.

**Lifestyles**	**PM_1**	***P***	**$PM_{2.5}$**	***P***	**PM_{10}**	***P***	**NO_2**	***P***	**Air pollution score**	***P***
**Smoking**		0.008		0.172		0.376		0.282		0.497
High	0.206(0.094, 0.318)*		0.449(0.315, 0.582)*		0.644(0.500, 0.788)*		0.712(0.549, 0.875)*		0.776(0.635, 0.918)*	
Low	0.077(-0.014, 0.168)		0.408(0.298, 0.519)*		0.663(0.544, 0.781)*		0.723(0.589, 0.857)*		0.809(0.700, 0.918)*	
**Drinking**		<0.001		0.002		<0.001		<0.001		0.001
High	0.501(0.245, 0.757)*		1.123(0.744, 1.502)*		1.007(0.670, 1.344)*		1.123(0.744, 1.502)*		1.073(0.734, 1.413)*	
Low	0.108(0.035, 0.182)*		0.697(0.590, 0.804)*		0.636(0.541, 0.731)*		0.697(0.590, 0.804)*		0.783(0.694, 0.872)*	
**Diet**		0.493		0.046		0.025		0.037		0.059
High	0.123(0.035, 0.210)*		0.384(0.279, 0.489)*		0.596(0.483, 0.709)*		0.655(0.528, 0.782)*		0.748(0.640, 0.856)*	
Low	0.185(0.065, 0.304)*		0.575(0.428, 0.722)*		0.839(0.682, 0.995)*		0.923(0.744, 1.102)*		0.939(0.796, 1.082)*	
**Physical activity**		0.529				0.505		0.741		0.646
High	0.172(0.040, 0.305)*		0.474(0.312, 0.636)*		0.714(0.540, 0.888)*		0.759(0.561, 0.956)*		0.832(0.667, 0.997)*	
Low	0.122(0.039, 0.206)*		0.421(0.321, 0.521)*		0.645(0.537, 0.752)*		0.719(0.598, 0.841)*		0.794(0.693, 0.895)*	

P, *P* for interaction; *, $P < 0.05$.

PM_1, particulate matter with an aerodynamics diameter ≤ 1.0 μm; $PM_{2.5}$, particulate matter with an aerodynamics diameter ≤ 2.5 μm; PM_{10}, particulate matter with an aerodynamics diameter ≤ 10 μm; NO_2, nitrogen dioxide. Concentrations of long-term exposure to four air pollutants were reflected by 3-year averaged concentrations before baseline of this study.

Adjusted for age, gender, marital status, educational level, personal averaged monthly income, smoking, drinking, diet score, physical activity, family history of CHD, family history of stroke, family history of T2DM, and family history of hypertension (unless stratified by the respective factor).

Multiplicative interaction of air pollution and healthy lifestyles score on cardiometabolic risk score was assessed by including the main effects of them and the product term in the model and the $P_{interaction}$ was represented by the p-value of product term.

Supplementary Table S12. The associations of ambient air pollutants (per IQR increment) and air pollution score (per IQR increment) with cardiometabolic risk score (β, 95%CI) by healthy lifestyle score.

Air pollutants	Healthy lifestyles score		
	T1	T2	T3
PM_1	0.230(0.123, 0.338)*	0.139(0.030, 0.248)*	0.013(-0.172, 0.199)
$PM_{2.5}$	0.513(0.385, 0.642)*	0.465(0.333, 0.597)*	0.423(0.195, 0.651)*
PM_{10}	0.754(0.615, 0.893)*	0.717(0.576, 0.858)*	0.688(0.448, 0.929)*
NO_2	0.825(0.668, 0.981)*	0.794(0.634, 0.954)*	0.759(0.484, 1.034)*
Air pollution score	0.901(0.765, 1.036)*	0.854(0.722, 0.985)*	0.824(0.613, 1.035)*

*, $P < 0.05$.

T: Trintile; PM_1, particulate matter with an aerodynamics diameter ≤ 1.0 μm; $PM_{2.5}$, particulate matter with an aerodynamics diameter ≤ 2.5 μm; PM_{10}, particulate matter with an aerodynamics diameter ≤ 10 μm; NO_2, nitrogen dioxide. Concentrations of long-term exposure to four air pollutants were reflected by 3-year averaged concentrations before baseline of this study. Adjusted for age, gender, marital status, educational level, personal averaged monthly income, family history of CHD, family history of stroke, family history of T2DM, and family history of hypertension.

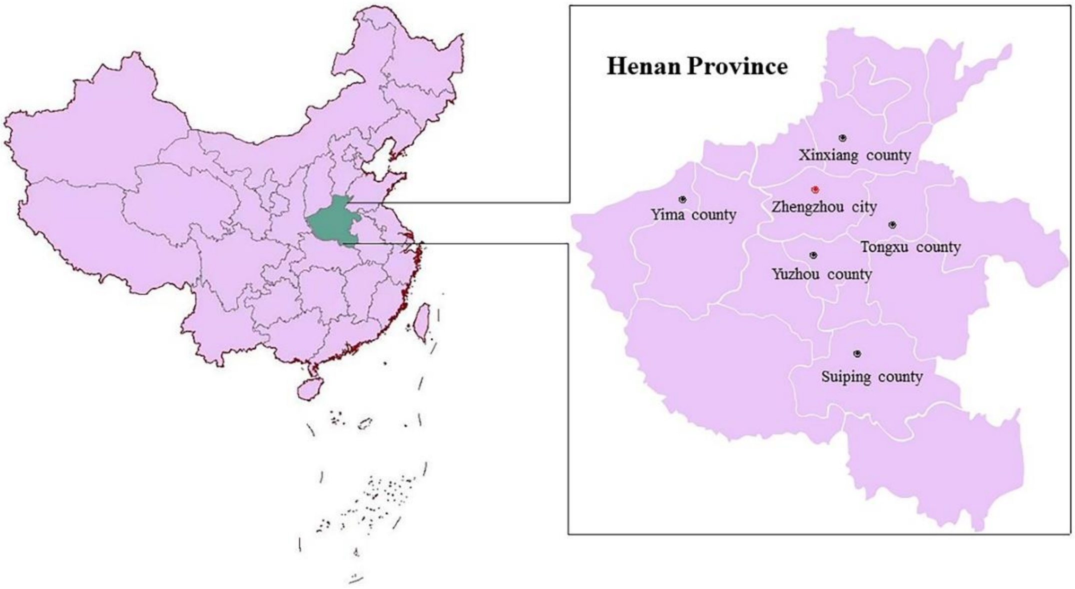

Supplementary Figure S1. Locations of the five survey sites in the Henan Rural Cohort.

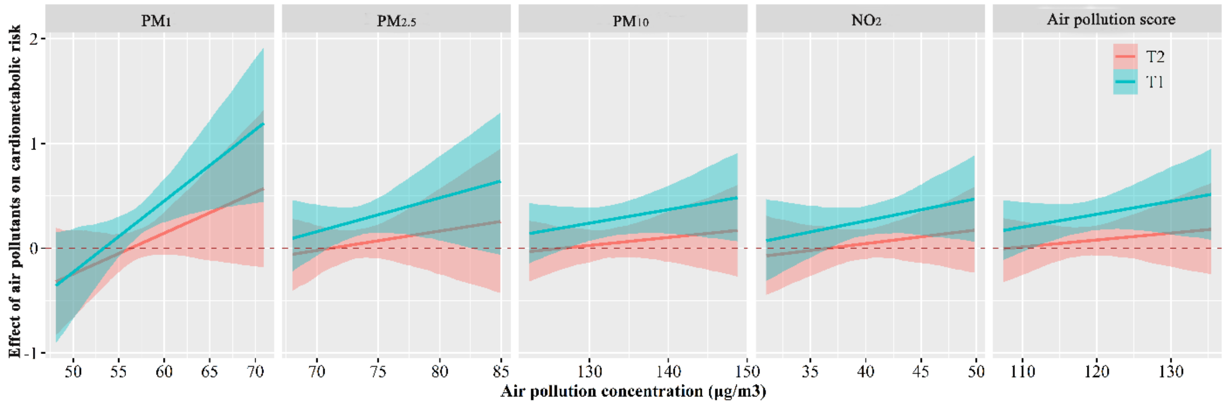

Supplementary Figure S2. Changes in estimated associations of healthy lifestyle score on cardiometabolic risk score along with increased levels of single- air pollutant and air pollution score. Highest tertile of healthy lifestyle score was used as the reference group. The models were adjusted for age, gender, marital status, educational level, personal averaged monthly income, family history of CHD, family history of Stroke, family history of T2DM, family history of hypertension. The lines and corresponding shaded areas represented the estimated effect and 95% confidence interval of healthy lifestyle score on cardiometabolic risk along with increasing levels of single- air pollutant and air pollution score. Concentrations of long-term exposure to four air pollutants were reflected by 3-year averaged concentrations before baseline of this study.

Estimating concentrations of air pollutants

In this manuscript, a machine learning method (random forests) was used to develop predicted models for concentrations of PM_1, $PM_{2.5}$, PM_{10}, and NO_2. Daily satellite-observed aerosol optical depth (AOD; from the MODIS satellite) and tropospheric NO_2 from the ozone monitoring instrument (OMI, level 3 NO_2 product) were used as independent variables in models to evaluate the concentrations of PMs (PM_1, $PM_{2.5}$, and PM_{10}) and NO_2. In addition to satellite-based data, meteorological (such as relative humidity, barometric pressure and wind speed) and land use data (percentage of urban cover and greenness) were also incorporated into the model to estimated air pollutants. The formula equations as follows:

$$PM_1 = AODc \times province + s(TEMP) \times province + s(RH) \times province + s(WS) \times province + s(BP) + firesmoke \times province + NDVI \times province + Forest\_cover + Urban\_cover + Water\_areas + month + Dayofweek + \log(elev)$$

$$PM_{2.5\ or\ 10ij} = AOD_{ij} + TEMP_{ij} + RH_{ij} + BP_{ij} + WS_{ij} + NDVI_{ij} + Urban\_cover_{ij} + doy_i + \log(elev_j)$$

$$NO_{2ij} = OMI_{ij} + TEMP_{ij} + BP_{ij} + RH_{ij} + WS_{ij} + NDVI_{ij} + Urban\_cover_{ij} + doy_i + \log(elev_j)$$

where $PM_{2.5or\ 10ij}$ represented the $PM_{2.5}$ or PM_{10} on day i at fixed station j; NO_{2ij} represented the NO_2 on day i at fixed station j; $AODc$ or AOD_{ij} exhibited the combined AOD; OMI_{ij} represented the satellite-derived OMI value; *province* represented the fixed station located in province; *TEMP*, *RH*, *BP* and *WS* indicated mean temperature, relative humidity, barometric pressure and wind speed on day i, respectively; *NDVI* represented the monthly average NDVI value at fixed station j; *firesmoke* represented the count of fire smoke spots; *Forest_cover* represented the percentage of forest cover (3-km radius buffer); *Water_areas* represented the percentage of water areas (10-km radius buffer); *Urban_cover* showed the percentage of urban cover with a buffer radius of 10 km around fixed station j; *doy* represented the day of the year; $\log(elev_j)$ meant the log transformed elevation.

References

Chen, G., Knibbs, L.D., Zhang, W., Li, S., Cao, W., Guo, J., et al., 2018a. Estimating spatiotemporal distribution of PM1 concentrations in China with satellite remote sensing, meteorology, and land use information. Environ Pollut. 233, 1086-1094. 18

Chen, G., Li, S., Knibbs, L.D., Hamm, N.A.S., Cao, W., Li, T., et al., 2018b. A machine learning method to estimate PM2.5 concentrations across China with remote sensing, meteorological and land use information. Sci Total Environ. 636, 52-60.

Chen, G., Wang, Y., Li, S., Cao, W., Ren, H., Knibbs, L.D., et al., 2018c. Spatiotemporal patterns of PM10 concentrations over China during 2005-2016: A satellite-based estimation using the random forests approach. Environ Pollut. 242, 605-613.
